# Factors that affect vaccines availability in public health facilities in Nairobi City County: a cross-sectional study

**DOI:** 10.11604/pamj.2021.38.72.21580

**Published:** 2021-01-21

**Authors:** Lucy Wanjiku Kanja, Peter Ndirangu Karimi, Shital Mahindra Maru, Pierre Claver Kayumba, Regis Hitimana

**Affiliations:** 1 East Africa Community Regional Center of Excellence for Vaccines, Immunization and Health Supply Chain Management (RCE-VIHSCM), University of Rwanda, Kigali, Rwanda,; 2 Department of Pharmaceutics and Pharmacy Practice, University of Nairobi, Nairobi, Kenya

**Keywords:** Cold-chain, facilities, immunization, logistics, Nairobi, stockout, subcounty, vaccine

## Abstract

**Introduction:**

over 1.5 million children die from vaccine-preventable diseases yearly. To avert these deaths and improve their livelihood, vaccine availability is important. The study assessed the availability of the vaccine, injection accessories and the associated factors in public health facilities in Nairobi City County and provided valuable data to contribute to improving healthcare infrastructure, stock management and vaccine distribution.

**Methods:**

a descriptive cross-sectional study was conducted in 68 randomly selected public health facilities at Nairobi City County in Kenya. Data was collected using a researcher-administered structured questionnaire and more information abstracted from the Vaccines management tools. The analysis was carried out using STATA version 14.

**Results:**

most facilities had experienced vaccines and accessories stock out at the time of the study and in the preceding twelve months. The most affected vaccines were tetanus (88%), measles-rubella (81%) and oral polio (79%). The causes of stockouts were rationing (82%), unavailability at the depot (93%), lack of transport (55%) and poor forecasting (50%). The majority (91%) of the facilities used the public transport system and only 1% had reliable government utility vehicles for delivery of vaccines and other logistics. Those near the vaccine depots preferred walking.

**Conclusion:**

the public health facilities in Nairobi City County experienced frequent stockouts of vaccines and accessories thereby exposing the residents to vaccine-preventable diseases.

## Introduction

Millions of children die yearly from vaccine-preventable diseases. To avert these deaths and improve their livelihood, the availability of vaccines is crucial. The ministry of health established the Kenya expanded programme on immunization in 1980, which was part of the global Expanded Programmes on Immunization (EPIs). The main goal was to control the killer vaccine-preventable diseases of childhood. There are four levels of vaccine movement in Kenya namely, national, regional, sub county and health facilities. To improve vaccine availability at the health facilities, the national immunization program policy recommends all sub county vaccine stores to have 3 months and 25% safety stock at all times. A study conducted in Kenya in the year 2014 showed that out of 300 sub-county vaccine stores across the country only 55% had an adequate cold chain storage [1]. This had implications on vaccine availability, as depots could not hold sufficient stocks. The report also revealed that although the national immunization program outsourced transport services for vaccine distribution between national and regional vaccine stores, there was no reliable transportation between regional and sub county vaccine depots as well as to the public health facilities. The mandate for transport and distribution to sub county and public health facilities lies with the county governments. The factors that affected vaccine availability included insufficient knowledge of forecasting, monitoring and resource management. Others were high staff turnover and wastage.

Kenya demographic and health survey (2014) report showed that only 79% of children had received all basic vaccinations between ages 12 - 23 months [2]. No country can achieve universal healthcare if its populations cannot access life-saving vaccines. Therefore, there is a need to continue assessing factors that affect vaccine availability in public health facilities and look for solutions. This will contribute to achieving the national vaccines and immunization program goal, which is to increase coverage to attain 90% at the national level and at least 80% in every district or its equivalent [3]. An effective vaccine management assessment conducted by the World Health Organization (WHO) and United Nations International Children's Emergency Fund (UNICEF) in conjunction with the ministry of health, showed that vaccines storage, distribution, stock management, temperature monitoring and lack of supportive functions performed poorly [4]. Immunization is considered to be among the most cost-effective investments in reducing morbidity and mortality due to vaccine-preventable diseases. However, vaccine availability, especially in developing countries, is still a challenge compared to developed countries, leading to deaths among children, which could otherwise be prevented [5]. Understanding factors that affect vaccine availability will help the government and partners supporting immunization services to plan and prioritize interventions based on gaps identified. This will improve access to immunization services to the mothers and children thereby reducing morbidity and mortality due to vaccine-preventable diseases. The objective of this study was to assess vaccine availability and associated factors in public health facilities in Nairobi City County.

## Methods

**Study setting and design:** this study was conducted in Nairobi City County; which is divided into ten sub counties to manage health care services. The delivery of these services and associated interventions are provided in four tiers. These include community, primary, county and tertiary levels. A descriptive cross-sectional study was carried out where 83 public health facilities provided the sampling frame. The sample size was determined using Fisher´s corrected formula for small populations. The multistage sampling method was used to select sixty-eight health facilities. The health facilities were initially categorized in strata according to the sub counties, the proportions were calculated and then simple random sampling done for selection.

**Data collection:** the data collection process started with a pilot study that was conducted in seven public health facilities, which accounted for 10% and were not among 68 sampled. The facilities were informed about the study and dates for the visit were scheduled. The respondents comprised of one participant per facility responsible for vaccine and immunization management who was either a pharmacist, nurse or clinical officer. Data collection was done in two approaches. The first approach was the use of researcher-administered structured questionnaires with closed-ended questions and Likert scale statements. The second approach-involved abstraction of data from vaccine forecast sheet, vaccines ledger books, vaccine order sheets, accessories counter requisition and issue voucher (S11), immunization summary booklet, cold-chain temperature monitoring sheets, inventory cards and stock cards. The physical count of the vaccine and accessories was conducted during the day of the study and results were compared with the minimum-maximum set limits as per the forecasting records for each facility. The adequate stock was considered if the physical count was within the minimum-maximum stock level requirement. Vaccines assessed were oral polio, measles-rubella, BCG, rotavirus, inactivated polio vaccine, DPT-HepB-HiB and pneumococcal. This was followed by an assessment of syringes, BCG (0.05ml), 0.5ml, 2ml, 5ml and safety boxes. On completion of the physical count, the respondent was asked whether there has been stock out of any vaccine or accessories for the last twelve months and the answers were counter-checked with vaccine ledger books, vaccine-ordering sheets records, accessories counter requisition and issue a voucher. If the answer was yes, the days out of stock were cumulatively added to know the actual duration. This was used as a means of verification of some of the answers given in the first approach responses. The duration of stockouts was categorized as from less than seven days to more than eight weeks.

**Data analysis:** data was coded and cleaned before the analysis. For easy management, data was captured in MS-Excel windows 2010. Analysis of the data was carried out using STATA version 14 and the results presented in tables and graphs.

**Ethical considerations:** approval to conduct the study was obtained from the University of Rwanda, Kenyatta National Hospital and University of Nairobi Ethical Research Committee (Ref: ERC- P220/03/2019) and Nairobi City County health research department (CMO/NRB/OPR/VOL1-2/2019/103).

**Informed consent:** participation in the study was voluntary and informants were free to withdraw or terminate during the interview. The researcher explained the nature of the study and ethical considerations therein before the interview. The respondents consented before the interview with assurance that the information shared was confidential and no names or identities were recorded.

## Results

**Sociodemographic characteristics:** the majority of respondents were female (98.53%) with nurses comprising 91.18%. Diploma holders were the majority (73.53%) with only 1% with master´s degree. On the duration of experience in the field of vaccine management, 33.82% had over five years and few (13.24%) had less than six months. Although the respondents had been working in the immunization areas for a while, only a few had attended the immunization training as shown in Table 1.

**Table 1:** sociodemographic characteristics (n=68)

Baseline characteristics	Variable	Number	Percentage
Gender	Male	1	1%
	Female	67	99%
Job title	Nurse	62	91%
	Clinical officer	6	9%
Education level	Certificate	8	12%
	Diploma	50	74%
	Degree	9	13%
	Master degree	1	1%
Vaccine management experience	<6 months	9	13%
	6 months to 1 year	8	12%
	1 - 2 year	10	15%
	>2 - 5 years	18	26%
	>5 years	23	34%
Expanded program on immunization training (operational or mid-level)	Yes	19	28%
	No	49	72%

**Figure 1:**
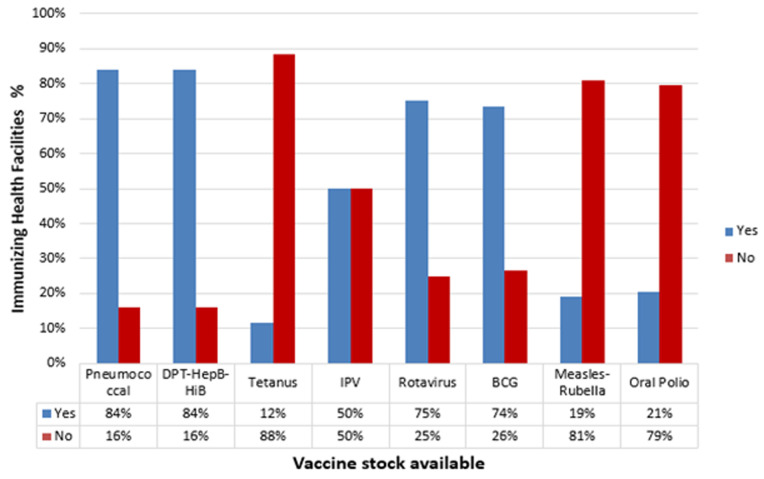
vaccines stock availability at the time of the study

**Figure 2:**
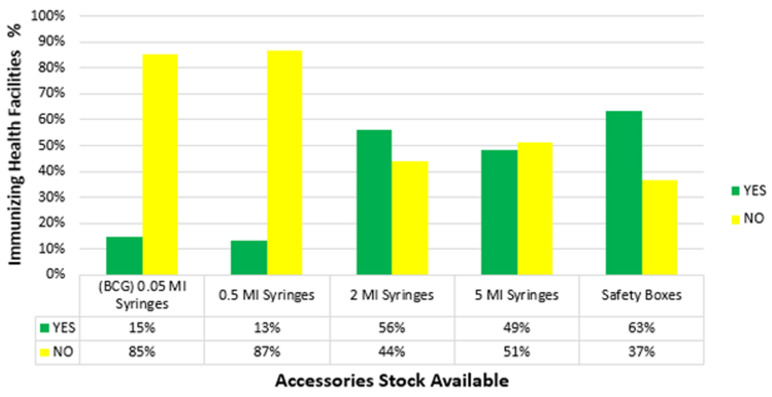
accessories availability at the time of the study

**Availability of vaccines and accessories:** most of the facilities had stock-outs of tetanus (88%), measles-rubella (81%) and oral polio vaccine (79%) as shown in Figure 1. For vaccines to be administered accessories should also be available. The majority of the facilities had stock out for 0.05ml syringes, 0.5ml and 5mls at 85%, 87% and 51% respectively. However, there was adequate stock of 2ml syringes (56%) and safety boxes (63%) as indicated in Figure 2.

**Vaccine stock status in the preceding 12 months:** the vaccine with the longest stock-outs period was tetanus, measles-rubella and oral polio lasting over one week to over eight weeks. Only 10% of facilities reported no stock-outs for tetanus, 9% for measles-rubella and 7% for oral polio vaccine. Pneumococcal and DPT-HepB-HiB had the shortest duration of stock-outs lasting less than seven days at 44% and 32% respectively as shown in Table 2.

**Table 2:** frequency of vaccines stock out in the preceding 12 months (n=68)

Type of vaccine	No of days stockouts (n,%)					
	No stockouts	<7 days	>1-2 weeks	>2-4 weeks	>4 -8 weeks	>8 weeks
Pneumococcal	31(46%)	30(44%)	5(7%)	0	2(3%)	0
DPT-HepB-HiB	46(68%)	22(32%)	0	0	0	0
Tetanus	7(10%)	11(16%)	8(12%)	12(18%)	28(41%)	2(3%)
IPV	15(22%)	24(35%)	23(34%)	4(6%)	2(3%)	0
Rotavirus	29(43%)	33(49%)	4(6%)	1(1%)	1(1%)	0
BCG	17(25%)	26(38%)	15(22%)	6(9%)	4(6%)	0
Measles-rubella	6(9%)	13(19%)	3(4%)	7(10%)	27(40%)	12(18%)
Oral polio	5(7%)	1(1%)	7(10%)	9(13%)	18(27%)	28(41%)

**Accessories stock status in the preceding 12 months:** prolonged stockouts of both 0.05ml (BCG) and 0.5ml syringes had been experienced (Table 3). Over 30% of the facilities reported stockouts lasting between four to eight weeks and 47% over eight weeks. Only 3% reported no stockouts of BCG syringes and 7% (5 facilities) for 0.5ml. This was attributed to stock out at the sub-county vaccine depot, poor forecasting, lack of knowledge on how to order and erratic supply from Kenya Medical Supply Authority (KEMSA). Twenty-four percent reported no stockouts for safety boxes.

**Table 3:** frequency of accessories stock out in the preceding 12 months (n=68)

Accessories	No of days stockouts (n,%)					
	No stockouts	<7 days	>1-2 weeks	>2-4 weeks	>4-8 weeks	>8 weeks
BCG syringe (0.05Ml)	2(3%)	4(6%)	1(1%)	8(12%)	21(31%)	32(47%)
0.5 Ml syringes	5(7%)	4(6%)	3(4%)	4(6%)	20(29%)	32(47%)
2 Ml syringes	15(22%)	13(19%)	11(16%)	14(21%)	8(12%)	7(10%)
5 Ml syringes	14(21%)	10(15%)	9(13%)	16(24%)	3(4%)	16(24%)
Safety boxes	16(24%)	33(49%)	11(16%)	1(1%)	1(1%)	6(9%)

**Figure 3:**
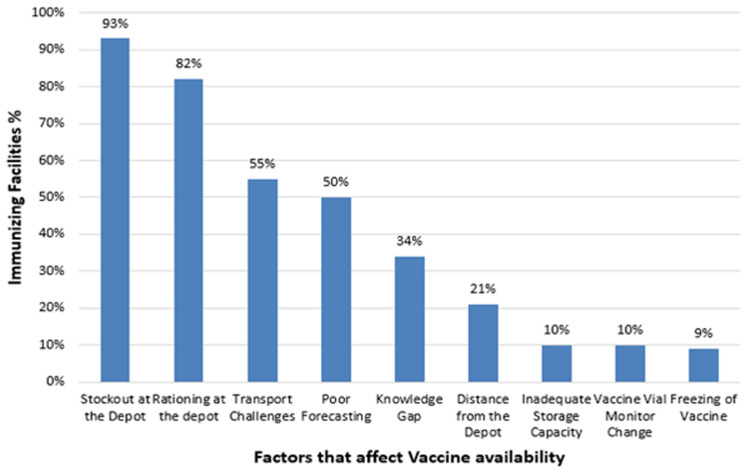
factors that affect vaccine availability (n=68)

**Factors that affect vaccine availability:** vaccine stock-out (93%) and rationing (82%) at the sub-county vaccine depot were major causes of the vaccine shortages (Figure 3). Transportation challenges accounted for 55% of the reasons precipitating the shortages. This was followed by poor forecasting (50%) and the knowledge gap on how to order vaccines at 34%. Distance from the vaccine depot, inadequate storage capacity and poor storage conditions, either due to un-recommended high or low temperatures rarely affected the vaccine availability.

**Transportation modalities:** the majority (91%) of the facilities used public transport system (buses, vans, taxis and motorcycles). Others used ambulances and those near the vaccine depots preferred walking. Only 1% of the facilities had a reliable government utility vehicle for use whenever they needed to collect vaccine and other logistics at the sub-county depot.

**Temperature monitoring assessment:** all facilities had 30 days temperature-monitoring tags (fridge tag 2E) with 96% having adequate storage capacity for they had received new cold chain equipment (refrigerators, vaccine carriers and icepacks). Although all facilities had a working thermometer stored with the vaccine, only 62% monitored the temperature all the times including weekends and public holidays. It was also noted that 53% reviewed the heat or freeze alarms and actions recorded. Over 40% did not have a contingency plan and a guide for the staff in case of emergencies or equipment failure. Only 10% of the facilities visited had a functioning generator connected as a backup in case of power failure.

## Discussion

Child vaccination is one of the most significant achievements in preventing infectious diseases and promoting child health. To improve vaccine access and immunization coverage uninterrupted supply of the right antigens, at the right place, right quantities, at the right time and in the right condition is critical. In this study, the results showed that the majority of facilities had adequate stocks of only three out of eight vaccines assessed. This is contrary to Kenya's immunization policy, which states that facilities should ensure there is the continuous availability of adequate stocks of all ministry of health procured vaccine, related immunological and support logistics at all levels of service delivery [3]. This also negates the global routine immunization strategies and practices coordinating actions to achieve disease prevention. For control of diseases like poliomyelitis, pneumonia, tetanus and measles. Supply chains and management should ensure the correct amounts of the right potent vaccine are available at each immunization session to enable children to receive the required vaccines on time [6].

Prolonged stock-out of tetanus, measles-rubella and oral polio vaccine, is a serious setback bearing in mind that these are vaccines preventing diseases that are targeted for elimination, control and eradication. Vaccine availability was hampered by stock out and rationing at the sub-county vaccine depot as well as transportation challenges. Proper planning of vaccine delivery and training of supply chain officers on vaccine handling to ensure vaccine maintain the potency are important [7]. In two studies conducted in Africa, some of the reasons for stockouts were the unavailability of the products at the vaccine depots and unreliable deliveries [8,9]. Extreme vaccine shortages were in line with those of stop stockouts national survey (SSP) conducted in South Africa [10].

On the duration of stockouts, Nairobi had a prolonged period with the majority having over four weeks. Vaccine stockouts are the main reason for incomplete immunization schedules and affect program image to the communities [9,11,12]. Prolonged shortage of accessories was also experienced. Kenya's health system is a shared responsibility between the national and county governments. The vaccine procurement process is through the national government whereas accessories are under county governments [1,13]. This was cited as a challenge since both systems are not well synchronized and stockouts of either affect the service delivery.

The majority of the facilities had WHO prequalified refrigerators and adequate storage capacity. Global routine immunization strategies and practices recommend regular capacity building and skills development for the health workers coupled with supportive supervision [7]. Training the staff on either operational or mid-level management is also important, coupled with mentorship and supportive supervision. Proper planning of vaccine delivery, training of supply chain officers on vaccine handling is essential to ensure that vaccines maintain their potency [7,8,14].

This study had some limitations as it focused on public health facilities only and excluded private facilities due to their nature of management. Secondly, the areas of interest involved were vaccine stock status, management and distribution as well as infrastructures supporting their availability. There was no assessment of the impact of stockouts on coverage and their contribution to vaccines preventable disease outbreaks. Of importance, to note is that since the study was conducted in Nairobi City County, which is an urban dwelling, the results may not be generalized to the rural setting. A renewed focus is critical to developing structures that will ensure effective vaccine management, good storage, transportation and delivery to the point of use safely and reliably. This commitment may lead to the growth of immunization programs by closing the existing gaps [14].

## Conclusion

The study shows vaccine and accessories stockouts occurred in public health facilities in Nairobi City County. The three grossly stocked-out vaccines were tetanus, measles-rubella and oral polio. Vaccine stock-out and rationing at the vaccine depots, transportation challenges and poor forecasting were the main factors associated with stockouts at the facility level. There is a need to have multisectoral collaboration from all stakeholders supporting immunization to harmonize the roles between national and county governments to improve vaccine availability in public health facilities.

### What is known about this topic

- To improve vaccine availability, access and immunization coverage uninterrupted supply of the right antigens, at the right place, in right quantities, at the right time and in the right condition is critical;
- Vaccine stockouts are the main reason for incomplete immunization schedules;
- Vaccine distribution is challenging compared to other commodities. This is because they must be stored and distributed at a certain temperature according to the manufacturer's instructions.

### What this study adds

- Importance of harmonizing supply chain for immunization in both county and national government to minimize interruption of service delivery;
- The need for intensive vaccine forecasting training and exercise at the sub-national level and health facilities for timely planning;
- The need to strengthen the collaboration of all stakeholders supporting immunization to harmonize the roles between national and county governments to improve vaccine availability.
